# Piezo1 as a potential player in intracranial hemorrhage: From perspectives on biomechanics and hematoma metabolism

**DOI:** 10.7555/JBR.37.20230241

**Published:** 2024-05-29

**Authors:** Tianle Jin, Maoxing Fei, Shiqiao Luo, Handong Wang

**Affiliations:** 1 Department of Neurosurgery, Nanjing BenQ Medical Center, the Affiliated BenQ Hospital of Nanjing Medical University, Nanjing, Jiangsu 210019, China; 2 Department of Neurosurgery, Nanjing Jinling Hospital, the Affiliated Hospital of Medical School, Nanjing University, Nanjing, Jiangsu 210002, China; 3 Department of Neurosurgery, the Affiliated Jinling Hospital of Nanjing Medical University, Nanjing, Jiangsu 210002, China

**Keywords:** biomechanics, hematoma, intracranial hemorrhage, Piezo1, secondary injury

## Abstract

Intracranial hemorrhage (ICH) causes numerous neurological deficits and deaths worldwide each year, leaving a significant health burden on the public. The pathophysiology of ICH is complicated and involves both primary and secondary injuries. Hematoma, as the primary pathology of ICH, undergoes metabolism and triggers biochemical and biomechanical alterations in the brain, leading to the secondary injury. Past endeavors mainly aimed at biochemical-initiated mechanisms for causing secondary injury, which have made limited progress in recent years, although ICH itself is also highly biomechanics-related. The discovery of the mechanically-activated cation channel Piezo1 provides a new avenue to further explore the mechanisms underlying the secondary injury. The current article reviews the structure and gating mechanisms of Piezo1, its roles in the physiology/pathophysiology of neurons, astrocytes, microglia, and bone-marrow-derived macrophages, and especially its roles in erythrocytic turnover and iron metabolism, revealing a potential interplay between the biomechanics and biochemistry of hematoma in ICH. Collectively, these advances provide deeper insights into the secondary injury of ICH and lay the foundations for future research.

## Introduction

Intracranial hemorrhage (ICH) is categorized based on pathogenesis as traumatic or non-traumatic ICH, or based on bleeding locations such as epidural, subdural, subarachnoid, or intracerebral ICH. Primary injury refers to the initial process leading to ICH, with a hematoma being the major consequence. Considering the biomechanics of trauma, traumatic ICH is considered having two main types of damage: focal damage (hematoma) secondary to contusion and diffuse damage (diffuse axonal injury) caused by accelerated or decelerated movements^[1–2]^. In traumatic ICH, brain tissues are deformed and impaired at the very moment of receiving direct external mechanical force to the head, followed by an instant hematoma, brain edema, and/or diffuse axonal injury. As the early dominant pathological manifestation, hematoma and brain edema cause an increased intracranial pressure that further alters the biomechanics, leaving remote tissues more vulnerable to ischemia^[3–4]^. In non-traumatic ICH, such as spontaneous intracerebral hemorrhage, vessels burst first, followed by the influx of blood into the brain parenchyma to form a hematoma, with an immediate secondary mechanical force occurring and being perpetuated because of the mass effect, rendering surrounding tissues deformed and damaged^[5]^.

Thus, the primary injury process of ICH is highly biomechanics-involved. However, previous studies mainly focused on biochemically-initiated alterations but made little progress. A few studies examined biomechanics-initiated downstream changes in ICH, but did not investigate specific factors involved in this process. Thus, a switch to biomechanics-initiated mechanisms might be more advisable. Coincidentally, Piezo1, a newly identified mechanics-gated receptor capable of mediating cation influx (Ca^2+^ mostly), is abundantly expressed in the membrane of various brain cells in vertebrates^[6]^, and may actively participate in the primary injury of ICH, providing a new perspective in the study of ICH.

After the primary injury mentioned above, individuals with traumatic or non-traumatic ICH undergo a similar pathophysiological process, termed secondary injury, during which lasting damage is exerted on intact brain tissues. Secondary injury is mainly triggered by cellular debris, blood-brain barrier (BBB) breakdown, and hematoma^[2]^, which may persist for hours to years and profoundly influence the prognosis. Thus, properly manipulating cellular and molecular events during the secondary injury process becomes extremely important. Several mechanisms are suggested to participate in the secondary injury, including neuroinflammation (a sterile immune reaction), cytoexcitotoxicity, oxidative stress, *etc.*^[7]^. Neuroinflammation plays a prominent role in this process, involving the activation of resident innate cells (*e.g.*, microglia and astrocytes) and the infiltration of peripheral blood components (*e.g.*, macrophages and neutrophils)^[7]^. Most of these mechanisms are, in fact, correlated, overlapped, and mutually complementary. Ultimately, they collectively contribute to the degeneration, necrosis (non-programmed cell death, nPCD), and programmed cell death (PCD) of cells, resulting in neurological pathology to different degrees.

**Figure 1:**
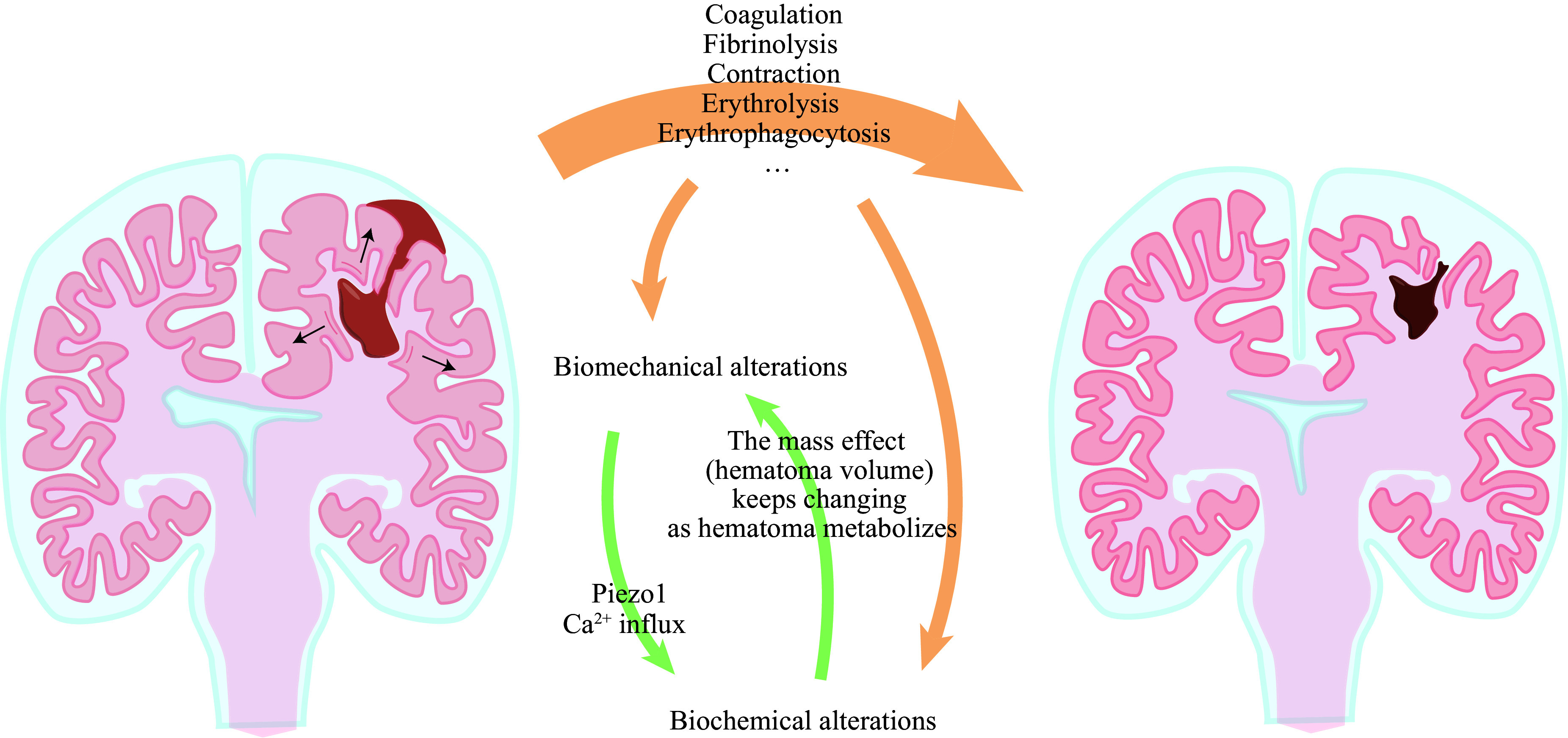
Piezo1 bridges the biomechanics with the biochemistry of hematoma in intracranial hemorrhage (ICH).

Hematoma, as the predominant pathological entity and the major pathogenic initiator of the secondary injury after the hyperacute phase, is the central topic of ICH. Comprising mainly of erythrocytes and plasma, hematoma metabolizes and undergoes pathophysiological changes related to the erythrocyte (erythrolysis^[8–9]^ and erythrophagocytosis) and the plasma (coagulation, fibrinolysis, and contraction) (***Fig. 1***). Along with these metabolic processes, both biochemical and biomechanical events occur. Specifically, as the hematoma metabolizes, biochemical events are triggered, resulting in changes in hematoma volume. The continuously changing hematoma volume results in an ever-altering mass effect, leading to variations in the biomechanical parameters of surrounding and some remote tissues. Cells may sense these biomechanical changes and subsequently convert mechanical cues into intercellular or intracellular signaling cascades (probably *via* Piezo1), through which a series of cellular and molecular events are triggered, leading to further pathophysiological alterations, such as astrocytosis that may change the biomechanics again.

In summary, with hematoma as the primary pathological condition, the secondary injury of ICH involves both biomechanics and biochemistry. Similar to the primary injury, Piezo1 likely plays a dynamic role in the secondary injury process. Therefore, in the following paragraphs, we will introduce Piezo1 and its potential roles in ICH in detail.

## The structure and gating mechanisms of Piezo1

Piezo1 is an evolutionarily conserved mechanically-activated cation (especially Ca^2+^) channel abundantly expressed in vertebrates and a large transmembrane protein with a molecular weight of approximately 286 kDa^[6]^. As a quite large homotrimer, Piezo1 consists of three identical subunits, each containing 38 transmembrane helices (TMs), a C-terminal domain (CTD), a C-terminal extracellular domain (CED) that homo-trimerizes to form the extracellular central cap (Cap), a beam, an anchor, *etc.*^[6,10]^, which makes Piezo1 a three-bladed propeller^[11]^. Specifically, Piezo1 is composed of three parts based on its configurational features and functional properties: the N-terminal mechanosensing module, consisting of three blades; the C-terminal pore module, conducting ions; and the transduction module, including the beam and anchor^[6]^. Each blade of the N-terminal module consists of 36 TMs (TM1–36), with every four TMs folded into one repetitive transmembrane helical unit (THU)^[6]^. Therefore, there are nine THUs composing one N-terminal blade. The blade exhibits a highly curved and nonplanar structure, resulting from membrane-parallel amphipathic helices that twist the lipid membrane into an inverted dome shape^[6]^. Adjacent to the central pore, TM37 and TM38 are termed the inner helix (IH) and outer helix (OH), respectively. A structure known as C-terminal OH–CED–IH–CTD, formed by an OH, a CED, an IH, and a CTD, homo-trimerizes to constitute the C-terminal pore module^[10]^. The beam bridges the distal blade to the central pore, with two ends, respectively, located in TM28 of THU7 and the CTD^[12]^. The anchor is composed of α1–3 helices, with α1–2 helices inserted into the gap between TM33 and OH, helping to retain the integrity of the configurations^[12]^.

**Figure 2:**
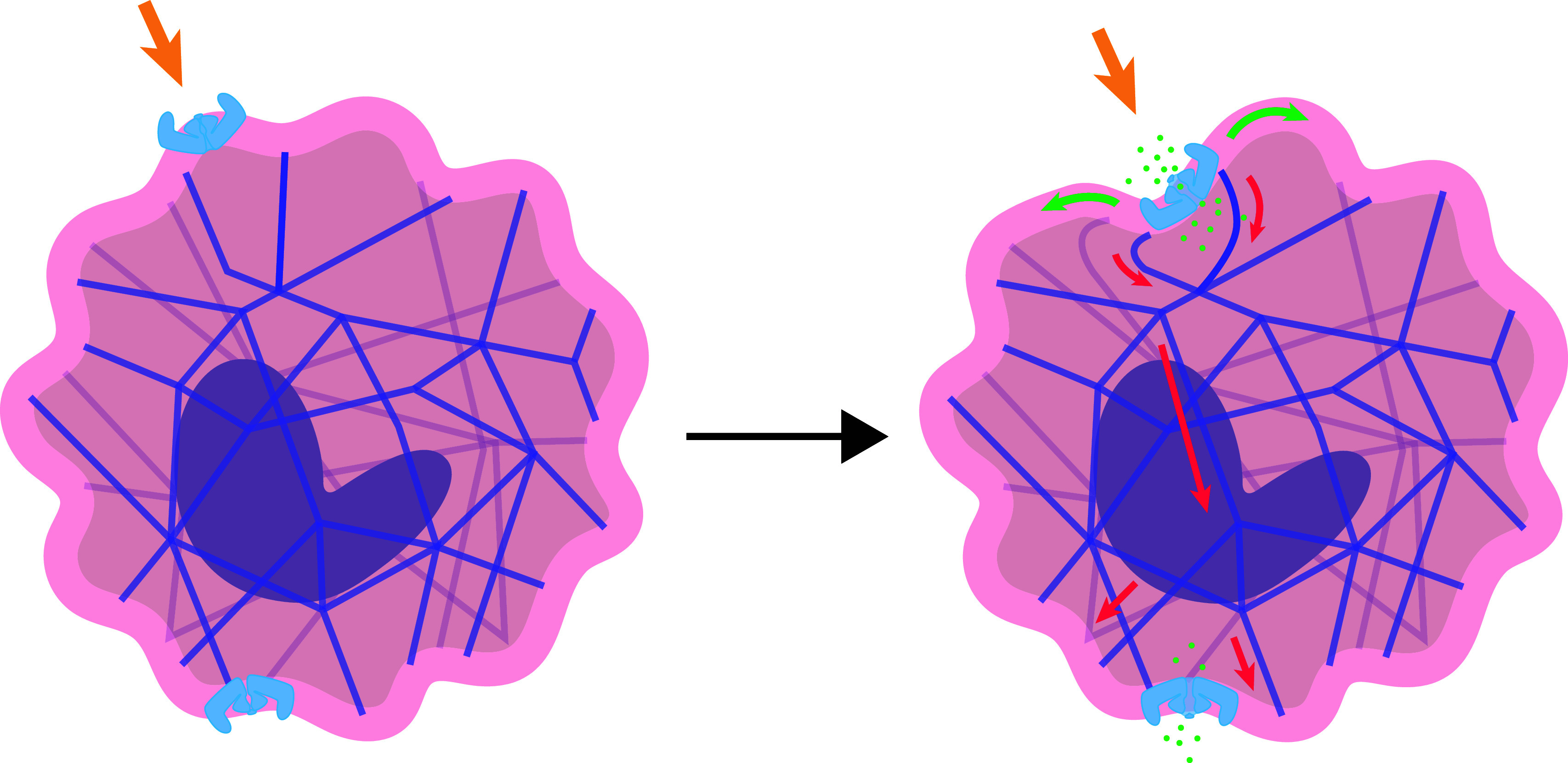
Piezo1 activated by mechanical force.

Two main well-received theories, namely the "membrane dome"^[13]^ and "membrane footprint"^[14]^, along with two principal models, the "force-through-lipid"^[15]^ and "force-through-filament"^[16]^, have been conceived to illustrate the force conduction process caused by mechanical stimuli. These theories and models are not mutually exclusive but rather mutually complementary, with the "membrane dome" theory significantly correlated with the "force-through-lipid" model. When receiving a mechanical stimulus, the lipid membrane deforms from its innate rigidity, causing previously curved blades to become planar^[13]^ (***Fig. 2***). Thus, the inverted-dome shape transforms with a tendency toward an even-plane conformation. Blades conduct the force to the central pore module *via* beams^[17]^, which consequently leads to the opening of the three lateral portals and the central TM pore enclosed by three IHs^[18]^. In a resting mode, the three lateral portals are blocked by the lateral plugs, and the TM pore is blocked by the central plug, with the lateral and central plugs fixed to the latch domain onto the central vertical axis, forming a sophisticated mechanical conduction system^[18]^. Upon sensing mechanical stimuli, the sealed gates will be unplugged, allowing cations to flux into the cell concurrently^[18]^. As a critical part of the pore module, the Cap domain on the TM pore, in response to mechanical stimuli, will develop an extracellular fenestration to allow the entry of cations into the TM pore^[19]^, which means that the Cap domain may play a crucial role in the TM pore gating. On the other hand, the "membrane footprint" theory is more related to the "force-through-filament" model. This theory indicates that Piezo1 activation occurs in response to remote mechanical vibration across the cell or internal forces generated within the cell, facilitated through the intracellular cytoskeleton system^[20]^ (***Fig. 2***). Still, many details about the structure and the gating mechanism of Piezo1 remain to be explored.

Upon the activation of Piezo1, the Ca^2+^ influx occurs and triggers the calcium signaling pathway and other overlapping pathways, resulting in further pathophysiological events.

## The roles of Piezo1 in the secondary injury

### Neuron and oligodendrocyte

The anatomical and functional integrity of neurons and oligodendrocytes is the main determinant of normal neurophysiological activity, and these two types of cells are significantly related both anatomically and functionally^[21]^. For instance, the injured axons contribute to demyelination, while demyelination may also initiate neurodegeneration^[22]^. Furthermore, neurons have a limited capacity to proliferate and repair themselves, making the process challenging^[22]^. The ongoing neuronal damage and loss can be observed in ICH. Thus, efforts to manipulate neuronal repair and myelination are profoundly meaningful. Insights into the roles of Piezo1 in neurons and oligodendrocytes in ICH may provide new strategies.

**Figure 3:**
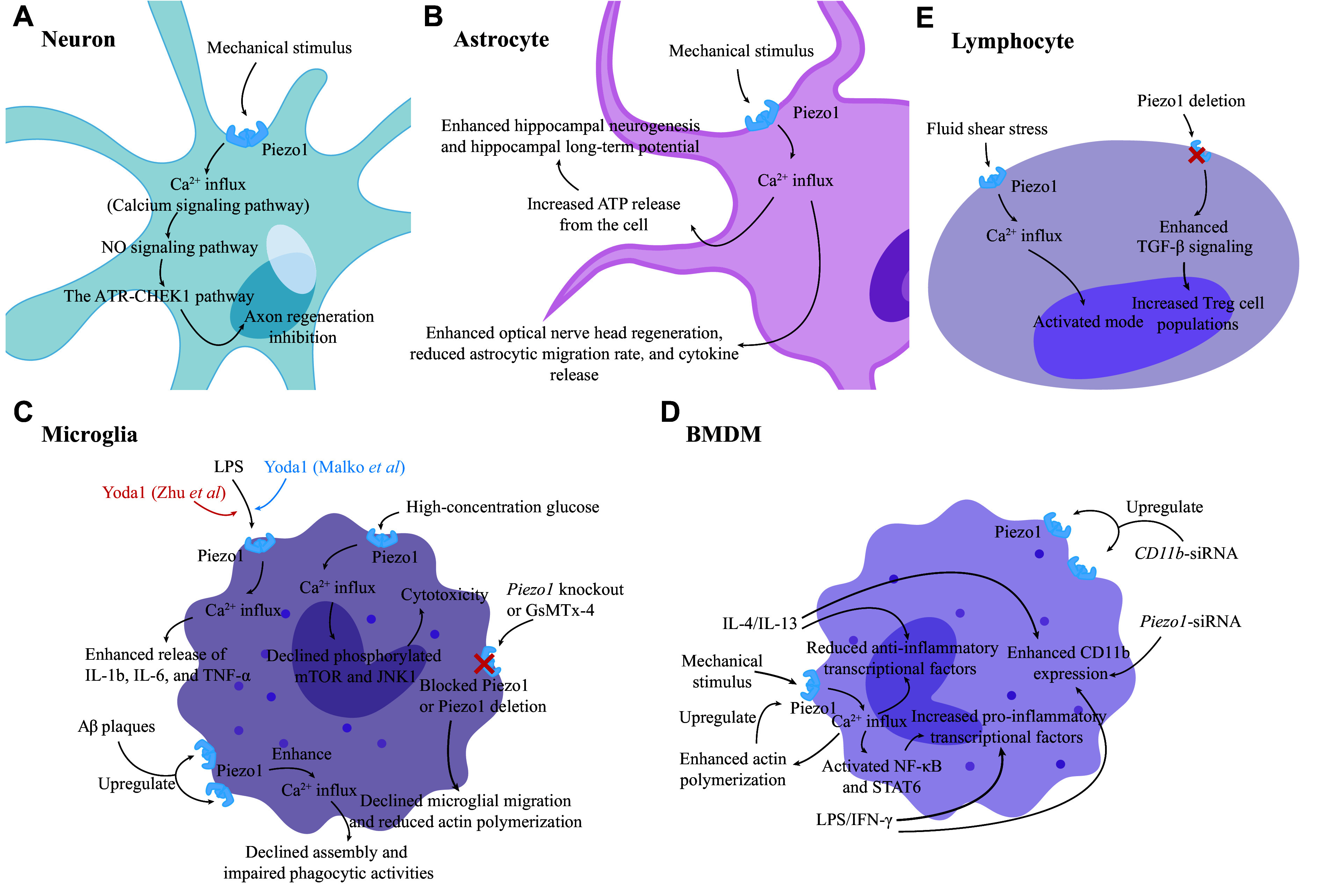
Mechanisms may be involved in the secondary injury of intracranial hemorrhage.

The ataxia telangiectasia and Rad3 related (ATR)-checkpoint kinase 1 (CHEK1) pathway is a significant barrier to neuronal repair and myelination^[23]^. In the past, investigators simply regarded this pathway as a DNA-damage-dependent response because of limited knowledge. This classic signaling pathway is summarized as follows: a single-stranded DNA activates ATR, which then phosphorylates CHEK1; the phosphorylated CHEK1 inhibits the phosphatases, cell division cycle 25C (CDC25C) or CDC25A, thereby impeding the dephosphorylation of cyclin-dependent kinase 1 or cyclin B and thus leading to a cell cycle arrest^[23]^. Axon regeneration has long been a hot topic in neuronal repair. Previous studies conducted on *Drosophila* revealed that the ATR-CHEK1 pathway negatively regulated the regeneration of sensory neuron axons and dendrites^[24–25]^. More recently, also in *Drosophila*, the ATR-CHEK1 pathway was shown to be activated independently of the damaged DNA, but rather by mechanical stimuli mediated by Piezo1 and its downstream nitric oxide (NO) signaling pathway, leading to the inhibition of axon regeneration^[26–28]^ (***Fig. 3A***).

Because Piezo1 gates Ca^2+^ influx and is highly expressed in neurons (but not in mature oligodendrocytes)^[29]^, it raises the question about what exactly happens to neurons, when other signaling pathways overlap with the calcium signaling pathway. Unfortunately, limited studies have been conducted to further explore other downstream signalings in the relevant scope up to now. However, similar experiments have been performed in other tissues or organs in various models. For example, a study in 2022^[30]^ demonstrated that in mice, mechanically-activated Piezo1 facilitated chondrocyte ferroptosis in osteoarthritis by increasing the Ca^2+^ influx and subsequently inhibiting GPX4, the key enzyme of ferroptosis. However, many downstream or even upstream mechanisms mediated by Piezo1 in neurons still need to be explored.

### Astrocyte

Astrocytes are one of the most abundant neuroglia in the brain and actively participate in diverse physiological and pathophysiological processes, including mechanical and nutritional support, electrical signal insulation, migration guidance for other cells, repair by astrocytosis, and antigen-presenting^[31]^. In the past decade, studies on astrocytes in ICH have mainly focused on biochemical-initiated signaling events, but there have been some advances in understanding the roles of biomechanics-triggered events mediated by astrocytic Piezo1.

The calcium signaling pathway plays an important role in the pathophysiology of astrocytes. A recent study showed a heterogeneous Ca^2+^ influx occurring spontaneously, specifically located in the processes of astrocytes in both awake mice and brain slices^[31]^. In 2022, Chi *et al*^[32]^ confirmed the expression of Piezo1 in the somas and processes of astrocytes in mice, and they uncovered that astrocytic Piezo1, responsible for the mentioned Ca^2+^ influx, could sense subtle mechanical cues and then transduce them into calcium signaling, ultimately triggering ATP release from astrocytes. Their subsequent studies indicated that astrocytic Piezo1 played a positive regulatory role in adult hippocampal neurogenesis *via* ATP-dependent proliferation of neural stem cells; additionally, by releasing ATP from astrocytes, astrocytic Piezo1 fostered the production of hippocampal long-term potentiation, which served as the mechanistic foundation of learning and memory, ultimately leading to the improved cognitive functions^[32]^ (***Fig. 3B***). Another study focused on optic nerve head astrocytes revealed that astrocytic Piezo1 was necessary but insufficient for astrocytic regeneration^[33]^ (***Fig. 3B***). Collectively, we believe that astrocytes, equipped with innate, well-designed subtle mechanical sensors in their bush-like processes where Piezo1 is located, are capable of developing an ever-large network (the astrocytic network formed by gap junctions). This network allows them to monitor microenvironmental biomechanical events, ensuring well-orchestrated physiological functions and responses against pathophysiological factors when necessary.

Other studies were conducted to investigate the interactions between astrocytic Piezo1 and neuroinflammation. One of these studies demonstrated that lipopolysaccharide (LPS) upregulated Piezo1 in cultured primary mouse cortical astrocytes in *vitro* and that the activated Piezo1 reduced the astrocytic migration rate and inhibited astrocytic cytokine release, including interleukin 1β (IL-1β) and tumor necrosis factor α (TNF-α), which alleviated astrocyte-related inflammatory cascades^[34]^. Another study in 2023 showed that LPS treatment sensitized Piezo1 of the mouse cerebral astrocytes and significantly promoted Piezo1-mediated Ca^2+^ influx, which further influenced (inhibited probably) the neuroinflammatory response^[35]^. All these advances have contributed to a deeper insight into the role of astrocytic Piezo1 in neuroinflammation, though more details about relevant mechanisms remain to be explored in future studies.

### Microglia

As resident innate macrophages in the brain, microglia are activated and arrive at the impaired foci soon after the primary injury. They are responsible for initiating and modulating inflammatory and recuperative responses to affect the central nervous system in a primary attempt to provide protection. The polarization of macrophages, including that of microglia, refers to the process by which macrophages are activated in a given temporal and spacial microenvironment, exhibiting a specific phenotype^[36]^. Activated macrophages are generally divided into two phenotypes termed M1 (classical activation) and M2 (alternative activation), and one phenotype may change into another in different settings^[36]^. However, this classification oversimplifies this dynamic process that contains a continuum of different intermediate phenotypes between pro-inflammation M1 and anti-inflammation M2^[37]^. Determining the factors that precisely affect the macrophage phenotype and manipulating relevant factors to attain beneficial phenotypes is of great clinical significance. However, similar to studies on neurons and astrocytes, past research on microglia has largely focused on biochemically-initiated alterations, with relatively less emphasis on biomechanically-caused events. Nonetheless, some Piezo1-related progress has been made in the past few years.

For example, Zhu *et al*^[38]^ demonstrated that functional Piezo1 was expressed in primary and BV2 microglia and that BV2 microglia treated with LPS and Yoda1 (a Piezo1-specific agonist) resulted in increased mRNA levels of *IL-1b*, *IL-6*, and *TNF-α*, compared with microglia treated with LPS only; they also demonstrated that LPS-treated mice with conditional *Piezo1*-knockout (KO) in microglia resulted in decreased expression levels of these cytokines; furthermore, both *Piezo1*-KO BV2 microglia and BV2 treated with GsMTx-4 showed declined migration rates, with the former accompanied by reduced actin polymerization levels^[38]^ (***Fig. 3C***). Microglial migration ability mediated by Piezo1 was later tested in mice with conditional *Piezo1*-KO microglia and chemoattraction assays, and the findings, consistent with results *in vitro,* indicated the decreased migration^[38]^. However, regarding the roles of microglial Piezo1 in modulating inflammatory cytokine release, Malko *et al*^[39]^ reached different conclusions. Instead, they found that microglial Piezo1 activated by Yoda1 inhibited the generation of LPS-induced microglial activation and pro-inflammatory cytokines (TNF-α and IL-6) in primary mouse microglia (***Fig. 3C***). Furthermore, in BV2 microglia cultured with high-concentration glucose (HCG)^[40]^, the inhibition of microglial Piezo1 was found to elevate the release of LPS-induced microglial pro-inflammatory cytokines (*e.g.*, TNF-α, IL-1β, and IL-6). These differences may result from the distinct properties between primary microglia and BV2 microglia, different modeling strategies, different approaches to quantify cytokine expression, and some unexpected factors. However, more experiments are required to conclusively confirm the exact effects and explain the distinction.

Nevertheless, some investigators shifted their focus to amyloid-β (Aβ) plaques. Aβ plaques are known for their prominent pathogenic role in Alzheimer's disease (AD) and have been associated with the occurrence of neurodegenerative disorders after primary injury, such as post-ICH AD and Parkinson's disease^[41–42]^. Hu *et al*^[43]^ revealed that microglial Piezo1 was upregulated under the stimuli of Aβ plaque stiffness in wild-type mice. They also observed a declined assembly of microglia, along with impaired phagocytic activities, in microglia-selective *Piezo1*-KO mice, which indicates positive and vital roles of microglial Piezo1 in mediating microglial sensing and clearing of Aβ plaques (***Fig. 3C***). Additionally, Piezo1 activation in familial AD mice was found to alleviate behavioral deficits with the improved hippocampal long-term potentiation; however, the same treatments to microglia-selective *Piezo1*-KO mice did not lead to identical positive feedback^[43]^. Similar conclusions were reached in studies by Jäntti *et al*^[44]^ as well.

High osmotic pressure is also a proper activator of Piezo1 by deforming the cellular membrane. Liu *et al*^[40]^ demonstrated that in BV2 microglia, HCG upregulated and activated microglial Piezo1 and that inhibition of microglial Piezo1 restored the levels of phosphorylated mammalian target of rapamycin (mTOR) and c-Jun N-terminal kinase 1 (JNK1) against HCG *via* the reduced Ca^2+^ influx, further protecting microglia from HCG (***Fig. 3C***). Therefore, the JNK1 and mTOR signaling pathways likely serve as downstream components of Piezo1, providing more manipulative points to investigate Piezo1.

### Bone-marrow-derived macrophage (BMDM)

Under the influence of certain cytokines and chemokines, BMDMs in systemic circulation are activated and recruited across the BBB to the damaged foci, occurring relatively later after the primary injury, where these BMDMs phagocytose cellular debris and metabolic waste and release various cytokines to further alter pathophysiology. BMDMs also displayed phenotype phenomena^[36]^. Several studies below about the interactions between Piezo1 and BMDMs may elucidate universal mechanisms that also apply to ICH. In 2021, by using transgenic mice, Atcha *et al*^[45]^ found that the mechanical activation of BMDMs was accompanied by Ca^2+^ influx exclusively mediated by Piezo1. The expression of macrophagic Piezo1 enhanced inflammatory responses to LPS/interferon γ (IFN-γ) but reduced healing responses to IL-4/IL-13 by upregulating pro-inflammatory transcriptional factors. Activation of these factors relied on intracellular Ca^2+^, including nuclear factor kappa-light-chain-enhancer of activated B cells (NF-κB) and signal transducer and activator of transcription 6 (STAT6). Furthermore, their study demonstrated an interplay, a positive feedback regulation, between the actin cytoskeleton and Ca^2+^ influx mediated by Piezo1 in BMDMs^[45]^. More specifically, the activated Piezo1 could cause Ca^2+^ influx that subsequently boosted macrophagic actin polymerization, and the enhanced actin polymerization in turn increased Piezo1 activities (probably sensitized Piezo1), ultimately leading to macrophagic pro-inflammatory responses^[45]^. Their another study^[46]^ provided more details, showing that both static and cyclic stretch suppressed LPS/IFN-γ-induced Piezo1 expression, leading to the decreased inflammation. No corresponding differences were observed in the unstimulated or IL-4/IL-13-treatment conditions. Moreover, they disclosed that both LPS/IFN-γ and IL-4/IL-13 elevated the expression levels of the integrin CD11b in BMDMs, and that static/cyclic stretch further increased expression levels of LPS/IFN-γ-induced CD11b, while cyclic stretch significantly decreased expression levels of IL-4/IL-13-induced CD11b^[46]^. A potential crosstalk between Piezo1 and CD11b was later shown, as evidenced by the observation that cells treated with *Piezo1* small interfering RNA (siRNA) increased CD11b expression, while cells treated with *CD11b* siRNA increased Piezo1 expression, compared with control BMDMs^[46]^ (***Fig. 3D***).

Toll-like receptor 4 (TLR4) and its downstream signaling pathways are essential for innate immune responses and the later development of adaptive immune responses against invaders. In the study of Geng *et al*^[47]^, it was confirmed that in BMDMs harvested from mice infected by *E.* *coli* and treated with LPS, both Piezo1 and TLR4 assemble to form complexes, and that Piezo1 might work as a co-receptor of TLR4 to influence related signaling pathways. Mechanistically, LPS stimulated TLR4 and the latter probably induced Piezo1 to mediate Ca^2+^ influx, which in turn activated calmodulin-dependent kinase Ⅱ (CaMKⅡ), Hippo kinases MST1 and MST2, and small GTPase RAC1, finally leading to actin cytoskeleton remodeling and enhanced phagocytosis in BMDMs^[47]^. This study provided a new insight into reconsidering the roles of Piezo1 in ICH by revealing the interplay between Piezo1 and TLR4.

In a dental study^[48]^, it was revealed that mechanically-activated Piezo1 fostered macrophagic proliferation exclusively *via* the PI3K-AKT pathway and Cyclin D1. Another study on bone formation^[49]^ demonstrated that BMDMs polarized to the M2 phenotype, increasing the secretion of transforming growth factor-β1 (TGF-β1) in a Piezo1-dependent manner. Although these findings were obtained from different tissues and organs, they offer the potential to enlighten future investigations on the roles of macrophagic Piezo1 in ICH.

### T-lymphocyte

According to the functional characteristics, T-lymphocytes are categorized into three types of effector cells: helper T (Th) cells, regulator T (Treg) cells, and cytotoxic T-lymphocytes (CTLs). As active regulators of the innate immune system and dominators of the adaptive immune system, T-lymphocytes profoundly impact various physiological and pathophysiological processes, including bacterial infections, sterile inflammation, wound healing, neoplasms, *etc*. Traditionally, there is a consensus that T-cell receptors (TCRs) of naïve T-lymphocytes recognize peptide-MHC (p-MHC) complexes presented by antigen-presenting cells (APCs) to exclusively initiate the activation of naïve T-lymphocytes and downstream immune events. However, more recent evidence has pointed out that mechanical cues of the T-lymphocytic micro-environment do influence the activation of naïve T cells.

One study showed that Piezo1 optimized the TCR signaling to activate naïve T-lymphocytes by boosting Ca^2+^ influx and calpain activation^[50]^, while another study also showed that fluid shear stress facilitated the activation of naïve T-lymphocytes, dependent on the Piezo1-mediated Ca^2+^ influx under mechanical stimuli^[51]^ (***Fig. 3E***). Additionally, Jairaman *et al*^[52]^ reported that functional Piezo1 was expressed in T-lymphocytes; although Piezo1 was unnecessary for homing, interstitial motility, proliferation and TCR-mediated calcium signaling of CD4^+^ T cells in mice, the deletion of Piezo1 in CD4^+^ T cells was found to specifically increase Treg cell populations *via* the enhanced transforming growth factor-β (TGF-β) signaling to restrain neuroinflammation (***Fig. 3E***). However, how Piezo1 affects the TGF-β signaling requires more exploration^[52]^.

Although studies on the roles of T-lymphocytes in ICH are not as extensive as those of microglia or BMDMs, accumulating evidence indicates that T-lymphocytes are also recruited *via* the impaired BBB and exert long-lasting double-edged immune effects on the brain in a relatively later phase of ICH-pathophysiology^[53–54]^. All these advances offer more hints for future studies of ICH by considering the roles of Piezo1 in T-lymphocytic immune inflammation.

### Erythrocytic turnover and iron metabolism

Erythrocytes in hematoma undergo metabolism through erythrolysis and erythrophagocytosis, triggering a series of biochemical events^[55]^. Erythrolysis refers to the release of cytotoxic substances from erythrocytes, which subsequently cause damage *via* downstream signaling pathways^[55]^. Both the complement system and eryptosis contribute to erythrolysis, with the former playing a more essential role^[55]^. However, it should be noted that eryptosis, as an erythrocyte-specific calcium-depend PCD pattern included in erythrolysis, is of potential importance and requires more insights^[56]^. Different from erythrolysis, erythrophagocytosis is a potent way to clear extravascular erythrocytes, conducted by microglia and macrophages, avoiding the release of cytotoxic components and interacting with adjacent cells, such as neurons^[55]^. However, the damage may still occur when aging macrophages are unable to return to the circulation system in time. Toxic components and phagocytes loaded with erythrocytes are not the end of the story; along with intact erythrocytes, they may be non-selectively transported out of the brain *via* cerebrospinal fluid (CSF) circulation pathways^[57]^. In addition to the well-known classic circulation pathway, where CSF is absorbed *via* arachnoid granulations back to the systemic circulation, the glymphatic system has come into focus in recent years as a powerful CSF clearance pathway but still is under debate as a newly proposed concept^[57]^.

The accumulation of ferrous/ferric ions (Fe^2+^/Fe^3+^) in intercellular micro-environments is a direct consequence of erythrocytic degradation, promoting consideration of its underlying effects on ferroptosis. Ferroptosis is an iron-dependent PCD pattern, and it occurs when intracellular Fe^2+^ is overloaded or anti-oxidant systems break down, leading to the production of reactive oxidative species *via* the Fenton reaction^[58]^. This process results in the peroxidation of polyunsaturated fatty acid chains in phospholipids to generate phospholipid hydroperoxides^[59–60]^, ultimately causing structural impairments and functional deficits in cells. The System x_c_^−^-glutathione (GSH)-glutathione peroxidase 4 (GPX4) pathway serves as the most powerful antioxidant system responsible for reducing phospholipid hydroperoxides against ferroptosis^[61]^. System x_c_^−^ is also a cystine/glutamate antiporter that mediates the uptake of cystine into cells to synthesize GSH, which is a vital cofactor of GPX4^[61]^. Intracellular overloaded Fe^2+^ results from a poorly-regulated cellular iron metabolism, involving either excessive inputs or insufficient outputs of iron. In general, extracellular Fe^3+^ may combine with transferrin to form complexes that are later accepted by the transferrin receptor, leading to the formation of endosomes and gaining access to the cytoplasm, while Fe^2+^ flows into cells directly *via* the zinc transporters, SLC39A8 (ZIP8) or SLC39A14 (ZIP14)^[62]^. Fe^3+^ inside endosomes is subsequently released from transferrin, then reduced to Fe^2+^ by six-transmembrane epithelial antigen of prostate 3 (STEAP3), and finally transported to the cytoplasm by divalent metal transporter 1 (DMT1) and ZIP8/14^[62]^. Free Fe^2+^ in the cytoplasm is specially termed labile iron pools^[63]^. Poly-r(C)-binding protein 1 (PCBP1) and 2 (PCBP1) are in charge of delivering free Fe^2+^ to apoproteins^[63]^, including ferritin (where Fe^2+^ oxidized to Fe^3+^ for storage) and ferroportin (where Fe^2+^ transported to the extracellular). Ferritin loaded with Fe^3+^ could develop autophagosomes, a process mediated by nuclear receptor coactivator 4^[64]^. These autophagosomes release Fe^2+^ under the influence of lysosomes rich in the reducing equivalents to satisfy the inner demand of cellular processes^[65–66]^. Thus, both intracellular Fe^2+^ and Fe^3+^ are capable of causing damage, with Fe^2+^ being more directly destructive as the culprit of the Fenton reaction. Cells after ferroptosis release abundant damage-associated molecular pattern signals that cause further damage.

**Figure 4:**
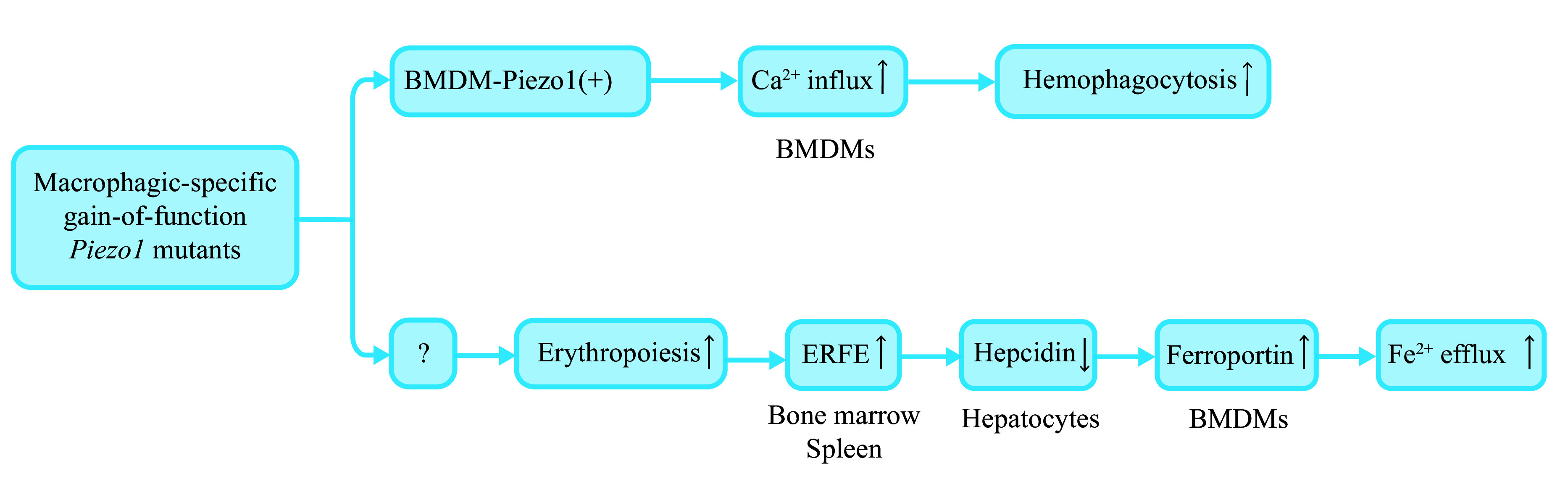
Erythrocyte turnover and iron metabolism in macrophagic-specific gain-of-function (M-GOF) *Piezo1* mutants.

Ardem Patapoutian, one of the laureates of the 2021 Nobel Prize in Physiology or Medicine, first revealed the role of Piezo1 in the iron metabolism of mice and humans^[67]^. In his study, he disclosed that overactive macrophagic Piezo1 (macrophage-specific gain-of-function [M-GOF] *Piezo1* mutations) facilitated enhanced erythrocytic phagocytosis of macrophages and consequent impaired hepcidin production, probably *via* the increased erythroferrone (ERFE) secretion; these changes ultimately resulted in iron overload and increased erythrocyte turnover in bodies^[67]^ (***Fig. 4***). Hepcidin is a protein hormone secreted by hepatocytes and participates in iron metabolism by contributing to the degradation of ferroportin, leading to reduced Fe^2+^ transportation to the extracellular environment^[68–69]^ (***Fig. 4***). ERFE, as a hormone produced from bone marrow and the spleen, on the other hand, downregulates liver hepcidin expression levels^[70]^. However, the upstream mechanisms that achieve upregulating ERFE when macrophagic Piezo1 is activated remain unknown^[67]^.

Based on the studies of Patapoutian *et al*, it is reasonable to hypothesize that in ICH, as the hematoma metabolizes, the ever-changing mass effect (hematoma volume) resulting from biochemical events may constantly alter the biomechanics of the hematoma itself and surrounding tissues, activating Piezo1 of various adjacent cells; the activated Piezo1, in turn, further impacts the pathophysiological processes of the hematoma and related tissues by increasing Ca^2+^ influx, possibly enhancing erythrocytic phagocytosis and iron exit of macrophages in/around the damaged areas (***Fig. 4***). Moreover, based on the discussion above, the increased Ca^2+^ influx into the cytoplasm and the elevated Fe^2+^/Fe^3+^ levels in the intercellular space may probably enhance ferroptosis in cells, causing more severe secondary damage. However, in the ICH situation, only macrophagic Piezo1 in peri-injured areas may be activated, which is quite different from M-GOF *Piezo1* mutations where macrophagic Piezo1 is overactive at the whole physical level. The real situation and the outcomes in ICH models lack potent experimental evidence, and much work is warranted to uncover universal underlying mechanisms that are manipulable to improve the hematoma-related brain damage in ICH.

## Conclusions and perspectives

From what has been discussed above, in one word, Piezo1 is a highly druggable target with a great clinical application potential. First, Piezo1 is a mechanically-activated cation channel located at the cellular membrane that mainly mediates the Ca^2+^ influx (calcium signaling pathway) to initiate downstream signaling, making it highly manipulatable *via* proper compounds. Second, the Piezo1-relevant mechanisms discussed above are actively involved in various pathophysiological processes (secondary injury) of ICH, and these processes are likely tightly related to the prognosis of ICH. Third, because the primary injury of ICH is inevitable, numerous previous studies were devoted to finding dominant biochemical-initiated mechanisms that led to the secondary injury of ICH, attempting to manipulate cellular and molecular events to ultimately improve the prognosis of ICH. However, in recent years, scarce new biochemical-initiated mechanisms have been identified, and a switch to mechanically-triggered mechanisms would probably be more advisable.

The studies mentioned above have revealed that Piezo1 is expressed in neurons, astrocytes, microglia, BMDMs, and T-lymphocytes, and undertakes corresponding physiological and pathophysiological roles in several specific models, including ICH ones. Specifically, the findings of Ardem Patapoutian *et al* theoretically uncover the potential Piezo1-dependent interplay between the biomechanics and the biochemistry of hematoma in ICH, implying the significant roles of Piezo1 in the pathophysiology of ICH^[67]^. It should be clarified that we should not leap to conclusions that Piezo1 is either helpful or harmful in physiological and pathophysiological processes and that the effects of Piezo1 vary in specific models. Thus, additional extensive research is necessary to elucidate the specific effects of Piezo1 on ICH and the underlying mechanisms in ICH models.
